# Tibial Osteotomy as a Mechanical Model of Primary Osteoarthritis in Rats

**DOI:** 10.1038/s41598-018-23405-3

**Published:** 2018-03-23

**Authors:** David Britzman, Ibidumo Igah, Theofano Eftaxiopoulou, Warren Macdonald, Anthony M. J. Bull

**Affiliations:** Department of Bioengineering, Imperial College London, South Kensington, SW7 2AZ UK

## Abstract

This study has presented the first purely biomechanical surgical model of osteoarthritis (OA) in rats, which could be more representative of the human primary disease than intra-articular techniques published previously. A surgical tibial osteotomy (TO) was used to induce degenerative cartilage changes in the medial knee of Sprague-Dawley rats. The presence of osteoarthritic changes in the medial knee compartment of the operated animals was evaluated histologically and through analysis of serum carboxy-terminal telepeptides of type II collagen (CTX-II). *In-vivo* biomechanical analyses were carried out using a musculoskeletal model of the rat hindlimb to evaluate the loading conditions in the knee pre and post-surgically. Qualitative and quantitative medial cartilage degeneration consistent with OA was found in the knees of the operated animals alongside elevated CTX-II levels and increased tibial compressive loading. The potential avoidance of joint inflammation post-surgically, the maintenance of internal joint biomechanics and the ability to quantify the alterations in joint loading should make this model of OA a better candidate for modeling primary forms of the disease in humans.

## Introduction

Osteoarthritis is a complex, multi-factorial disease which has both mechanical and biological effects on the whole synovial joint. It is characterized by severe damage to load bearing regions of articular cartilage, excess bone formation at the edges of the joints (osteophytosis), alterations to the morphology and structure of the subchondral bone, synovitis, and often inflammation or thickening of the joint capsule itself^1^. The disease is often separated into two distinct subtypes: primary and secondary, with secondary OA disease occurring as a result of a known affliction such as an Anterior Cruciate Ligament (ACL) rupture. Primary OA occurs mainly in elderly patients, developing gradually over time, and represents 82% of OA patients^2^. Because of the complex, multi-tissue nature of the disease it has proven hard to isolate the exact causes for degenerative changes in primary OA; however, it is likely that the cause is multi-factorial. It has been found that the progressive degeneration of the cartilage stimulates chondrocyte production, which helps to repair the tissue. When the process of cartilage destruction is faster than the attempted repair, further erosion and cracking of the cartilage ensues, leading to damage in deeper layers of the cartilage and even subchondral bone^2^. This destruction of the cartilage is caused by enzymes called matrix metalloproteinases (MMP’s) which are produced by synovial cells and chondrocytes. Inhibition of these proteins is an area under investigation for the development of new anti-arthritic medications^3^. Mechanical factors have long been known to play a role in the development of the disease^4,5^ but the extent to which alterations in natural mechanical loading impact upon osteoarthritic development is not yet fully elucidated. Recent studies have also postulated a large inflammatory aspect to the disease^6–8^.

Because of the difficulty in recruiting patients during the early, asymptomatic phase of disease pathogenesis, a variety of animal models have been developed to attempt to model the disease. The most commonly used methods tend to be surgically induced either using ACL transection (ACLT)^9,10^ or a medial meniscal tear (MMT)^11,12^. Both of these models involve surgical destabilization of the joint, leading to altered internal loading conditions and eventually OA. The issue with these models is that they are fundamentally models of secondary OA and as such their ability to provide translational benefits for primary OA patients is not necessarily clear. The intra-articular surgery itself can often lead to unintended damage to other important joint tissues, potentially triggering unwanted inflammatory responses^13^. Pure primary models of OA have tended to rely either on genetically inducing biological changes to increase the incidence of disease or utilizing a small subset of animals that develop OA spontaneously such as the Hartley guinea pig^14^. Studies looking at the histopathology of Hartley guinea pig knee joints at 18 months and 3 years old found biochemical changes which reflected reasonably well with those present in human patients^15^. The downside of these models, however, is that the long disease development time alongside relatively low incidence rates of the disease make them unsuitable for any large scale therapeutic studies^16^.

In order to avoid these issues, a number of non-invasive rodent models of post-traumatic disease have been developed^17^. These include cyclic tibial compression^18^, controlled external joint loading^19^ and intra-articular fracture of subchondral bone^20^. One methodology that has received fairly little interest of late is the tibial osteotomy model, whereby the tibia is reset at a varus (or valgus) angle to increase the mechanical loading on the medial (or lateral) side of the knee joint. This avoids the need for intra-articular surgery or destabilization of internal joint mechanics, potentially allowing the model to be used as an induced model of primary disease. This methodology is potentially more analogous to human primary OA, where alterations in external loading caused by malalignment of the tibia are known to lead to OA without any internal damage to the joint itself^21^. This is backed up by the fact that surgical interventions to realign the tibia have been shown to reduce or inhibit the development of OA on the medial side of the knee^22^. Previous models showing the pathogenesis of OA in response to surgically induced tibial angulation have mainly focused on the rabbit, as its large size (relative to rodents) simplifies the surgical procedure. In these cases angles of up to 30 degrees valgus have been used to overload the lateral tibial compartment and have been shown to lead to mild degenerative changes in the cartilage suggestive of early stage OA 12 weeks post-surgically^23,24^. The lack of substantive interest in these models can be partly related to the species chosen. Practical considerations have led to the decision to use larger mammals, however, the biggest demand for models of OA is in rodents, due to their low price, low space requirements and ease of use^25^. The exact effect that changing biomechanics have on the medial loading are also unknown in these studies, making it hard to translate the results to a clinical context. In this paper a novel model of primary OA in rats is proposed based on a surgically induced varus tibial malalignment. Alongside this, a biomechanical model is used to evaluate the levels of knee joint loading pre and post-surgery to quantify the specific alterations in local joint biomechanics that lead to medial knee OA.

## Results

### Histological Grading

**Figure 1:**
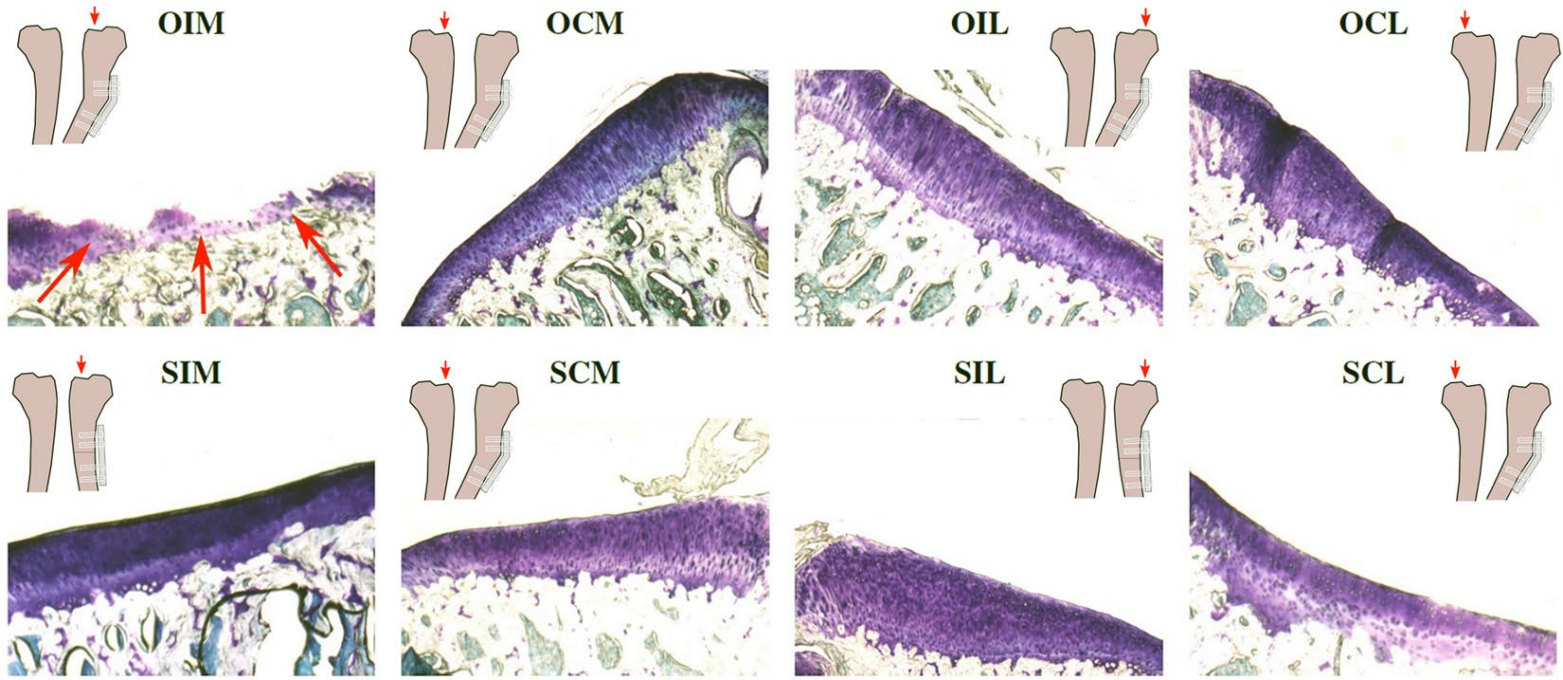
Representative toluidine blue stained sections of medial and lateral tibial plateaus for the contralateral and ipsilateral knees of the osteotomized animals with cartilage stained dark blue/purple. Osteoarthritic lesions present where the line of cartilage is disrupted. Site of severe lesions marked with red arrows. (OIM: Osteotomy Ipsilateral Medial; OCM: Osteotomy Contralateral Medial; SIM: Sham Ipsilateral Medial; SCM: Sham Contralateral Medial; OIL: Osteotomy Ipsilateral Lateral; OCL: Osteotomy Contralateral Lateral; SIL: Sham Ipsilateral Lateral; SCL: Sham Contralateral Lateral).

**Figure 2:**
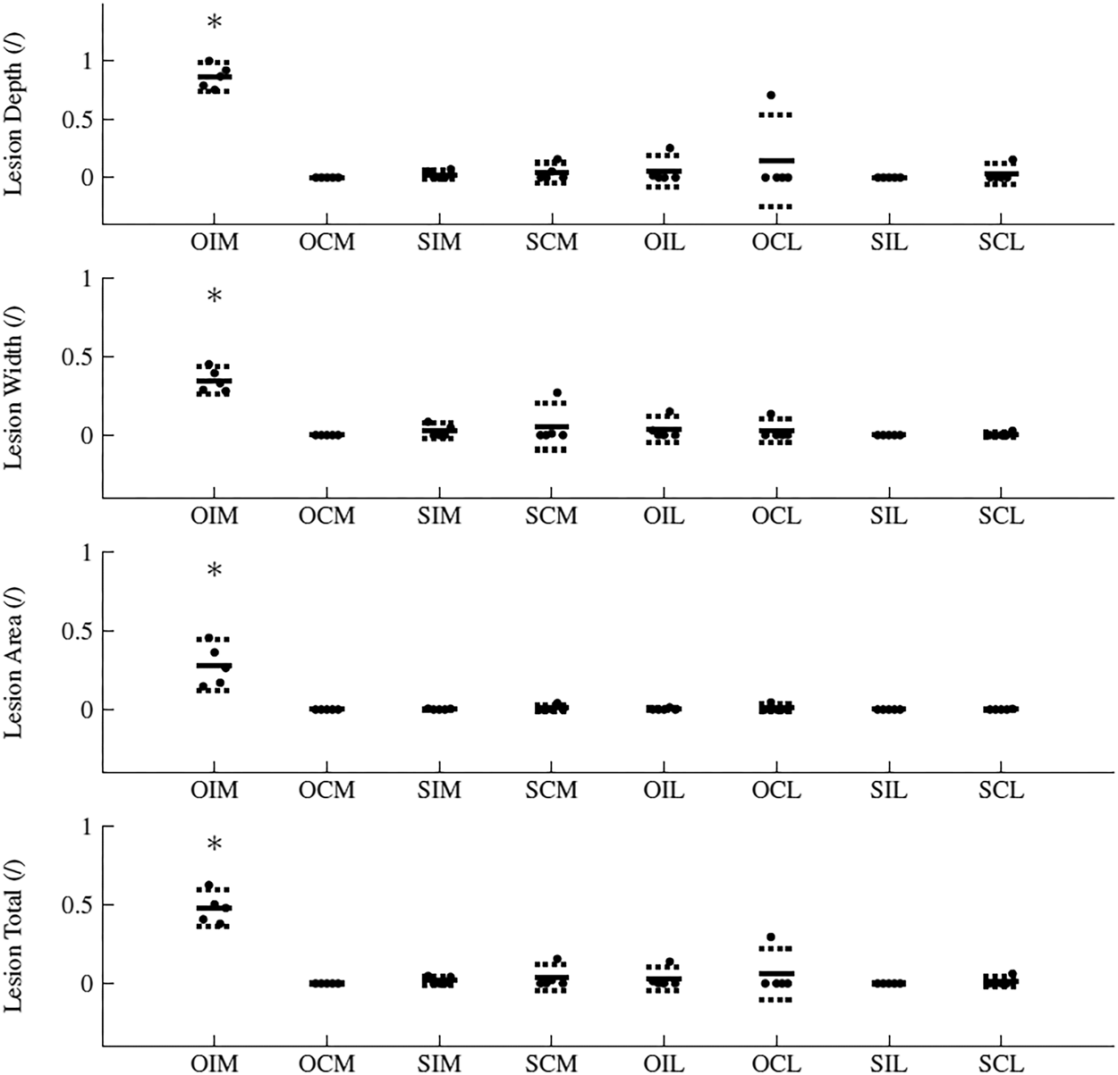
Lesion morphology in sham (n = 5) and osteotomized (n = 5) subjects for contralateral and ipsilateral limbs as per OARSI guidelines. Solid lines represent the mean and dashed lines represent the 95% CI. Asteriks denote significant difference at the 0.05 significance level between highlighted group and one or more of the other groups. (OIM: Osteotomy Ipsilateral Medial; OCM: Osteotomy Contralateral Medial; SIM: Sham Ipsilateral Medial; SCM: Sham Contralateral Medial; OIL: Osteotomy Ipsilateral Lateral; OCL: Osteotomy Contralateral Lateral; SIL: Sham Ipsilateral Lateral; SCL: Sham Contralateral Lateral).

Extensive cartilage matrix loss can be seen in the ipsilateral tibia of the osteotomized animals with severe lesions present (Fig. 1). Sham animals appear healthy, with a smooth lining of cartilage. Quantitative histological analysis confirmed the presence of degrenerative cartilage loss (Fig. 2). The one-way MANOVA determined that histological grading was statistically dependent on the eight sample types listed (p < 0.01). Post-hoc tests confirmed that there were significant differences between the ipsilateral medial knee of the osteotomized animals and all other parameters for lesion width (p < 0.01), lesion depth (p < 0.01), lesion area (p < 0.01) and total score (p < 0.01).

### Biomarker Analysis

**Figure 3:**
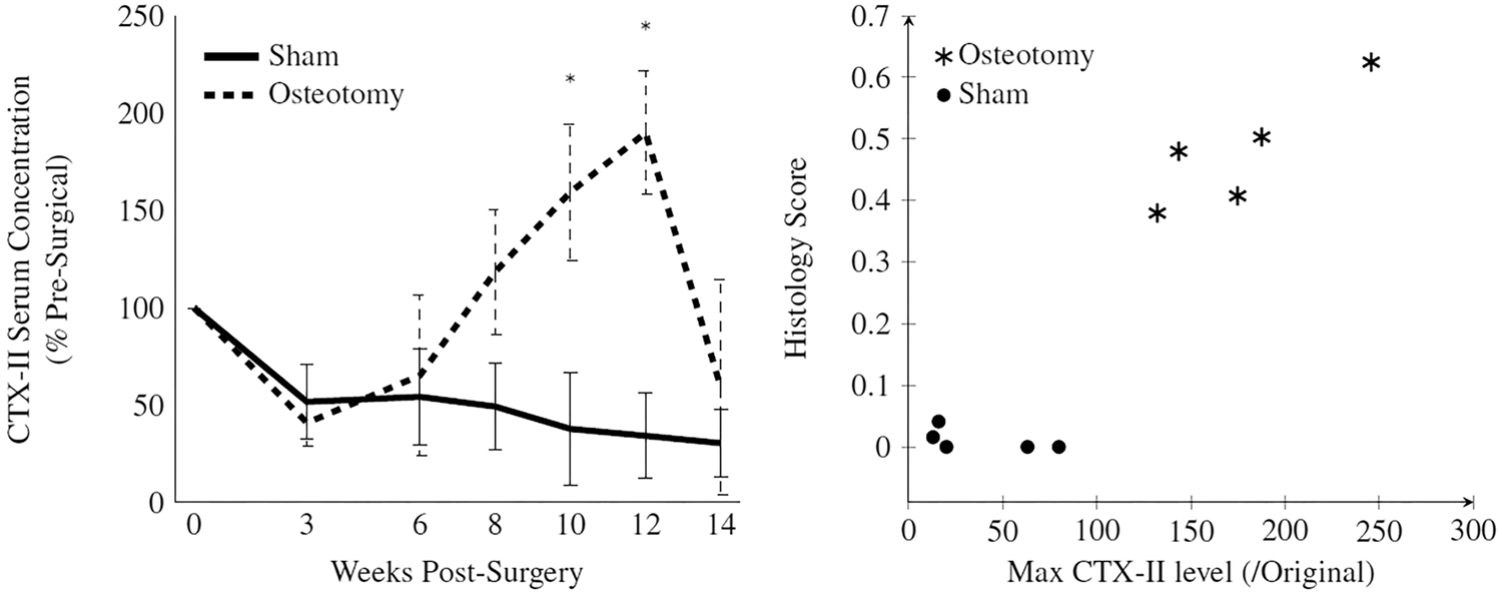
(Left): Serum CTX-II levels over the course of the experiment for both sham (n = 5) and osteotomized (n = 5) animals. Samples taken pre-surgically, 3 and 6 weeks post-surgically and then every 2 weeks subsequently until 14 weeks. Points plotted +/−SE with * indicating a significant difference at p < 0.05; (Right): Scatter graph showing the correlation of maximum serum CTX-II levels with end of study histological scores for sham and osteotomized animals.

For sham animals, CTX-II levels appear to drop over time, eventually reaching a plateau at around 40% of their original level (Fig. 3). Serum levels for the osteotomized group appear to follow the same trend for the first 3–6 weeks before jumping sharply between week 6 and 12 to 194% of their original level and then dropping off in week 14. There was a statistically significant interaction between the type of surgery performed and the time on the relative CTX-II serum concentration (p < 0.01). Post-hoc analysis of simple main effects revealed that the CTX-II level in the osteotomized groups was significantly higher than the sham subjects 10 and 12 weeks post-operatively (p < 0.01, p < 0.01).

### Biomechanics

**Figure 4:**
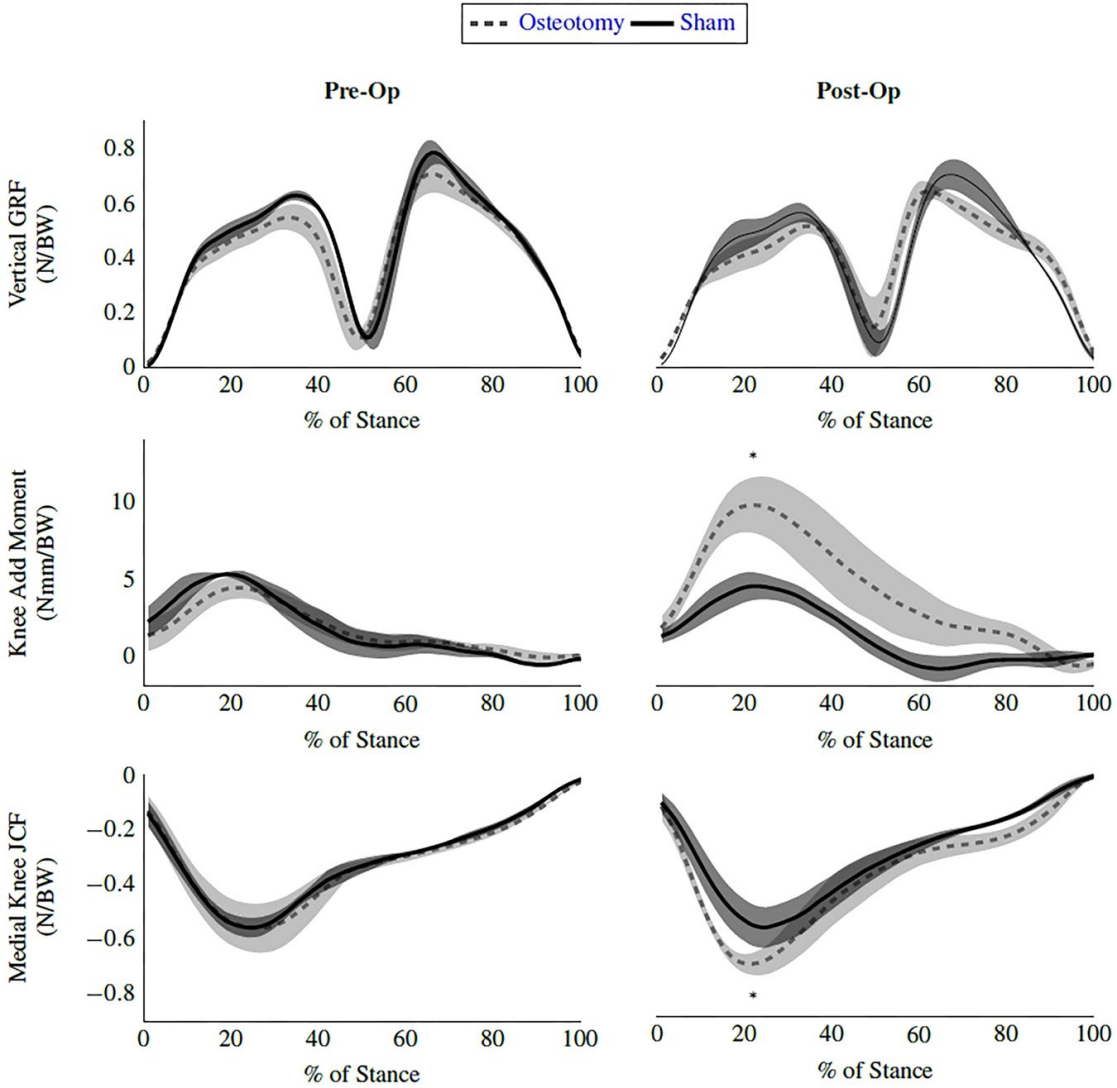
Biomechanics of the lower limbs pre surgically and 12 weeks post-surgically for sham (n = 5) and osteotomy (n = 5) animals. All forces reported as a fraction of bodyweight. Error bars +/−SE. *Denotes significant difference between sham and osteotomy samples at p < 0.05.

There was a statistically significant interaction between the type of surgery performed and time on both the medial knee joint contact force (JCF) (p < 0.01) and the knee adduction moment (p = 0.028) (Fig. 4). Post-hoc tests revealed that both the peak knee adduction moment (p = 0.011) and the medial knee JCF (p < 0.01) were higher in the osteotomized animals post-operatively, with no significant changes seen between the groups pre-operatively. Ground reaction forces post surgically in the osteotomized group seem to be consistent with both those measured pre-surgically and those of the control subjects, with no significant interaction found. This suggests the animals are still loading their limbs fully post-surgically, even with the presence of severe cartilage erosion.

## Discussion

Evaluating the exact pathophysiology of OA is difficult for a number of reasons, however, findings from recent animal work have proposed a dual role for both local joint biomechanics and systemic inflammation type activities^6,26,27^. Previous surgical models of the disease in rats have tended to involve intra-articular surgery which may lead to an increase in localized synovial inflammation and provide a misleading picture of how the disease initiates and spreads in primary disease patients. This paper has proposed a model in rats which could help to isolate the effects of biomechanical changes, quantify them and evaluate the impact they have on disease progression. Histological analyses showed that inducing a varus osteotomy in the animals produced severe cartilage matrix loss in the medial ipsilateral knee which was not present in sham animals. Quantitative analysis of the histological sections further highlighted the osteoarthritic cartilage changes present in the ipsilateral limb of the osteotomized animal, with lesions found to be bigger, wider and deeper than in both the contralateral limb and the sham animals. The level of disease seems to be significantly greater than that reported in previous studies using similar methods in rabbits^23,24^, however, due to the different species and varying husbandry conditions it is hard to provide an accurate comparison of the two models. One potential reason for the increased rate of development could be the species of animal chosen, with smaller animals having thinner cartilage and thus developing more substantial lesions at an earlier time point. Compared to many ACLT models, the rate of disease progression appears to be fairly gentle, with histological changes seen in rats subjected to ACLT in as little as 1 week post surgically^28^, whereas serum biomarkers in this study seem to suggest that osteoarthritic changes commence later. Further histological timepoints will need to be taken in future studies to evaluate whether this is the case. If so, then this would be beneficial in giving the ability to study the early phase of the disease over a longer time period and evaluate early stage interventions for which there would not be adequate time in other surgical models.

Vertical ground reaction forces pre and post surgically did not show any significant differences, which is an encouraging sign that the animal is loading its ipsilateral limb fully post-surgically. This optimizes the chances of the animal developing osteoarthritis through biomechanical mechanisms and should allow for good repeatability of the model. Analysis of knee joint contact forces show that maximum medial joint loading and adduction moments were significantly higher in the osteotomized animals. Previous studies in humans have demonstrated the link between these two measures and the incidence of OA^29^. One benefit of this model is that the biomechanical alterations made to the local environment can be easily adjusted by either changing the angle or placement of the osteotomy. By either increasing the angle of osteotomy or creating the tibial wedge higher up on the tibia the medial loading can be increased further, potentially inducing more rapid lesion development and cartilage degeneration. Adversely reducing the angle could have the opposite effect, and potentially allow for easier studying of early stage disease and preventative therapies. This gives great flexibility in how the model can be used; future studies will hope to elucidate this property more thoroughly. One limitation of this study, as presented, is the fact that tibial malalignment was not measured pre-surgically or monitored throughout the timescale of the study. This would give a better indication as to what degree the tibial malalignment was altered by the surgery and allow for correlations to be made between changes in tibial alignment and biomechanical loading at the knee. Serial micro-CT imaging in small rodents is becoming more practical due to the availability of small scale, high resolution scanners and could be used in future studies to monitor changes in tibial geometry through the experimental time period. Alongside serial histology of the knee, this may give a clearer indication of how anatomical variations in tibial alignment influence the progression of mechanically induced osteoarthritis.

CTX-II biomarker analysis has also served to validate the model, with levels of the biomarker rising after 6 weeks for the osteotomized animals and falling consistently throughout the study for the sham animals. CTX-II is known to increase significantly in human OA patients and is one of the best options currently available for non-invasive diagnosis of early stage disease^30^. In this study we were not able to evaluate how the timing of CTX-II changes corresponded to osteoarthritic changes as histology was done at 14 weeks only. However, a strong correlation between CTX-II levels and the level of histological cartilage degradation suggest that it may well be a good diagnostic and prognostic biomarker for OA in this model. This corresponds to work done previously which suggests that CTX-II can not only predict the presence of osteoarthritic changes, but also the severity of these changes. The fact that it falls sharply in the osteoarthritic animals towards the end of the study suggests that in the later stages of the disease, the biomarker may lose its ability to differentiate between healthy and diseased tissue as the serum level reverts back to normal. This has been shown in previous studies and highlights the difficulty in finding a consistent biomarker for osteoarthritis, where the disease evolves and changes rapidly, and different phases of the disease can have significantly different biological symptoms^31^.

The ability of the model to generate osteoarthritic changes without the need for intra-articular surgery has advantages. The problem with surgical models that operate on the joint itself is that it is unclear whether they are truly modeling the primary or secondary form of the disease. Recent animal studies have started to show clear pathogenic differences between primary and secondary forms of the disease, however, comparative studies of different model types within the same group are still very rare. A study by Wei *et al*.^32^ showed significant differences in serum biomarker levels of several cytokines known to be related to the development of OA. Lubricin, a glycoprotein present in synovial fluid that aids lubrication, was shown to significantly decrease in animals with ACLT induced OA compared to those with spontaneous disease development and control subjects. It is known from genetic studies that a reduction in lubricin levels significantly increases the rate of osteoarthritic development^33^ and it is known to decrease after traumatic knee injuries such as ACL rupture where a rapid inflammatory response is initiated in the affected knee^34^. Studies on synovitis post-injury have proposed lubricin as a key biomarker and the lack of a decrease in this marker in the spontaneous subjects could suggest that inflammatory responses occurring in the knee in post-traumatic disease models may not accurately reflect what happens in human subjects. Because of the overuse of these secondary’ models relative to the incidence of primary and secondary disease in humans, the role of inflammatory agents versus biomechanical changes may well be misunderstood in primary disease and intervention based on reducing or preventing this inflammatory element may not have the desired outcome. If it is true that the two diseases are fundamentally different in both origin and pathophysiology then it seems there is a need for two discrete sets of models. The model presented here has shown it is able to produce reliable, controllable, unicompartmental osteoarthritis without the need to open the joint capsule itself and run the risk of systemic inflammation, unnatural internal joint biomechanics or localized tissue damage. One way to test whether the disease mechanism is truly different in this biomechanical model of disease compared to existing intra-articular methods would be to investigate whether gender influences disease development. In this study male rats were chosen due to the higher incidence of disease reported in previous intra-articular models of OA when compared to female subjects^35^. The reason for the reduced incidence rate of OA in female subjects is thought to be due to the protection that estrogen and other female hormones provide against certain inflammatory disease pathways^36^. It is possible that in purely mechanical models of disease, as proposed here, inflammation is not the key driver of disease development and so the hormonal protection seen in female subjects may be reduced.

In summary, this paper has shown the clear effect that changing knee biomechanics has on degenerative cartilage loss and has presented a novel approach to altering these biomechanics in a controlled and quantifiable manner via the use of biomechanical modelling.

## Methods

### Animal Ethics and Approval

This study was performed under full institutional and departmental license with ethic committee and UK Home Office approval and all methods were performed in accordance with the relevant guidelines and regulations. Euthanasia was performed under license by overdose intraperitoneal (IP) injection of pentobarbitone. Animals were provided food and water ad libidum except where specific exemptions were approved under a license provided by the UK Home Office.

### Power Analysis

In order to avoid unnecessary use of animals, a power analysis was carried out prior to the study to evaluate minimum sample sizes for each group based on quantified histological damage seen in MMT operated animals in the literature^37^. The power analysis indicated that a minimum of 4 animals in each group was required to obtain 90% power to detect significant differences at the 0.05 level of significance.

### Animal Selection, Husbandry and Training

Ten healthy male Sprague-Dawley rats (12 weeks old) were allocated into two groups, one group of five to undergo varus osteotomy, and one sham group of five. The animals were placed in quarantine for 1 week prior to being handled, during which time cheerios (Nestlé, Vevey, Switzerland) were provided daily to familiarize the animals. The animals were then trained for 2 weeks to traverse a 1.5 m long walkway (FIG) following the guidelines set out by Webb *et al*.^38^. Throughout this time the animals were restricted to maintenance energy requirements to prevent excessive weight gain and to allow the cheerios to provide an incentive to train. At the end of 2 weeks any animals which could not successfully traverse the runway were culled using an appropriate schedule 1 method. In total 22 animals were used: 10 for the trial, 8 were culled post-training and 4 were culled post-surgically (1 due to removal of sutures, 2 due to bone fracture during surgery and 1 due to lameness in the ipsilateral leg post-surgery). Animals were kept in groups of 2–3 in 1500 cm^2^ individually ventilated cages throughout the study, except for one week post-surgically where they were single caged to allow for effective recovery and monitoring.

### Surgical Osteotomy

In order to increase loading on the medial compartment of the knee, surgical osteotomies were performed on 5 of the rats. Briefly, the animals were anaesthetized using isoflurane inhalation (induced at 5%, maintained at 2%) and administered pre-operative Buprenorphine (0.05 mg/kg) and Baytrill (10 mg/kg) via intraperitoneal injections (IP). The animal’s hind limb was clipped liberally and prepared for surgery. A 35 degree closing wedge osteotomy was created just distal to the knee joint and the two tibial sections manoeuvered to the correct surgical angle. A 25 mm long malleable plate with five 1.5 mm holes (Veterinary Instrumentation, Sheffield, UK) was bent to the angle of osteotomy and placed adjacent to the osteotomy so that four 1.5 mm holes could be drilled into the lateral side of the tibia. 1.5 mm self tapping cortical screws (Veterinary Instrumentation, Sheffield, UK) were used to attach the plate to the tibia and bring the closing wedge together to promote bone healing. Buprenorphine (0.05 mg/kg) and Baytrill (10 mg/kg) were provided once and twice daily respectively via IP injection for five days postoperatively. Sham animals were prepared for surgery as above and the animal was osteotomized (0 degree osteotomy) just distal to the insertion of the patella tendon, at the same level as for the varus osteotomy described previously. As above, a metal plate was used to provide fixation and the same post-operative rehabilitation was provided. The surgical procedure is displayed photographically in Supplementary Figure S1.

### *In-Vivo* Kinematics and Kinetics

**Figure 5:**
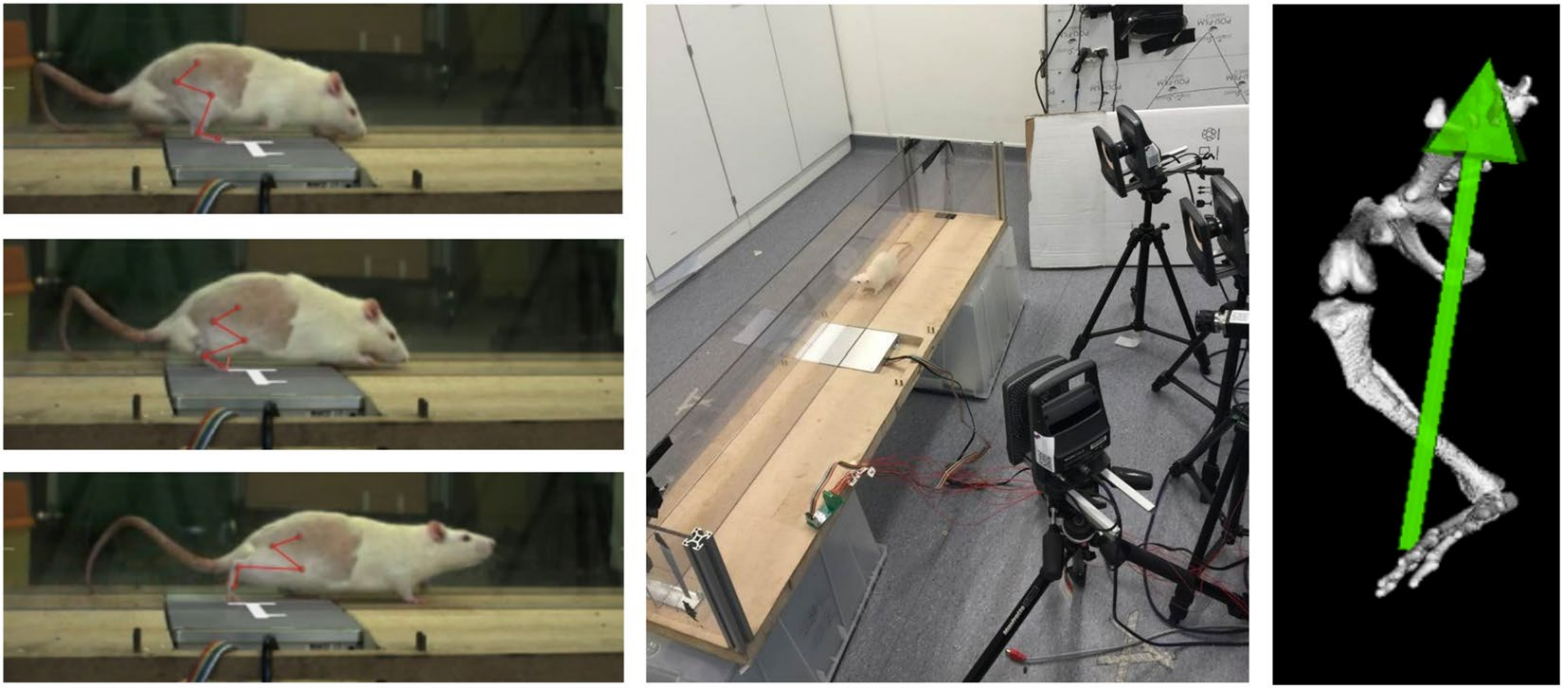
Experimental method for collecting *in vivo* kinetic and kinematic data (left). OpenSim model of the ipsilateral hindlimb of an osteotomized animal (right).

*In vivo* kinematics and ground reaction force (GRF) measurements were collected from the animals pre-operatively and then 6 and 14 weeks post operatively. 24 hours prior to data collection the animals were anaesthetized using inhalation anaesthesia (isoflurane 2% in O_2_), shaved liberally around both hind limbs and then marked using a non toxic marker at the locations of the 5th metatarsal, lateral malleolus, lateral femoral epicondyle, greater trochantar and articular rim^39^. On the day of experimentation 3 mm reflective markers were attached to the animals at the pre-marked locations and they were made to traverse a 1.5 m long perspex enclosed walkway using Cheerios as an incentive. An AMTI Hex6 × forceplate (AMTI Inc., MA, USA) was used to capture the GRF data as well as three Vicon T40 cameras (Vicon Motion Systems Ltd, Oxford, UK) positioned on the lateral side of the animal to detect the 3d locations of the reflective Markers (Fig. 5: left). Data was processed using vicon Nexus (Vicon Motion Systems Ltd, Oxford, UK) and exported to a standard motion format (C3D).

### Musculoskeletal Model

An existing musculoskeletal model of the rat hindlimb^40^ coded within OpenSim^41^ was used to determine the joint loading conditions post surgically (Fig. 5: right). Briefly, an extra fixed joint was created in the anterior portion of the tibia to replicate the point of osteotomy. This joint adducts about the tibia at a fixed angle which can be determined using subject specific post-culling measurements of the osteotomy angle. Body segment parameters for the two tibial segments were found via a least squares optimization approach such that the peak knee joint reaction force with an osteotomy angle of 0 degrees was as close as possible to that of the existing model. Inverse kinematics, inverse dynamics, static optimization and joint reaction analyses were used in OpenSim in combination with the *in vivo* measurements to determine the medial knee joint contact forces and adduction moments.

### Histology

At the end of the study animals were culled via schedule 1 methods appropriate for rats. The angle of osteotomy was recorded by exposing the tibia and then the knee was dissected, leaving 10 mm either side of the joint to avoid excess joint trauma. The patella was then removed and the knee was processed for histology using the protocol set out in Supplementary Table S1. Sections were taken every 50 *μ*m from the anterior section of the knee and stained every 100 *μ*m using toloudine blue. Sections were scored for lesion width, depth and area using OARSI guidelines. A total cartilage damage score was calculated by taking an average of the lesion width depth and area scores^42^.

### Serum Analysis

Serum samples were collected from the sham and osteotomized animals following ASPA guidelines pre-operatively as well as 3, 6, 8, 10 and 12 weeks post-operatively. Enzyme based assays were used to evaluate the serum levels of CTX-II (RatLaps TM, IDS, Boldon, UK) as per the manufacturer guidelines.

### Statistical Methods

Quantitative histology comparisons were generated using a one-way MANOVA to compare the difference in cartilage degeneration metrics between the eight groups shown in the qualitative histology images. Post-hoc univariate ANOVAs were carried out for each damage metric to evaluate the simple main effects where differences were detected. Statistical analyses of serum biomarker levels and biomechanical measures were completed using two-way mixed ANOVAs to consider both the effect of treatment group and of time, with each biomechanical parameter being considered separately. Post-hoc univariate ANOVAs were undertaken to investigate where statistically significant differences between the osteotomized and sham groups occurred. Where appropriate, tests for normality (Shapiro-Wilk) or sphericity (Machly) were conducted prior to each analysis. All analyses were carried out using IBM SPSS Statistics for Windows, version 24 (IBM Corp., Armonk, N.Y., USA).

### Data Availability

The data that support the findings of this study are available from the corresponding author upon reasonable request.

## Electronic supplementary material

Supplementary Information
