# Supplementary material for: Tibial Osteotomy as a Mechanical Model of Primary Osteoarthritis in Rats
Source: Sci Rep. 2018 Mar 23;8:5132. doi: 10.1038/s41598-018-23405-3 (PMC5865111; doi:10.1038/s41598-018-23405-3)
Supplement: Supplementary file 1 — Supplementary Information [file 41598_2018_23405_MOESM1_ESM.pdf]

Supplementary Information for: Tibial Osteotomy as a Mechanical Model of Primary Osteoarthritis in Rats

David Britzman[1,*], Ibidumo Igah[1], Theofano Eftaxiopoulou[1], Warren Macdonald[1], and Anthony M J Bull[1]

[1]Department of Bioengineering, Imperial College London, South Kensington, SW7 2AZ, UK
[*]david.britzman12@imperial.ac.uk

Table S1. Histological protocol for processing of rat knees

Step	Chemical	Duration	Notes
Fixation	10% NBF	48 hrs	Change every 24 hrs
Decalcification	10% Formic Acid	21 days	Change every 24 hrs
Dehydration	50% Ethanol	1 hr	
Dehydration	60% Ethanol	1 hr	
Dehydration	70% Ethanol	1 hr	
Dehydration	80% Ethanol	1 hr	
Dehydration	90% Ethanol	1 hr	
Dehydration	100% Ethanol	1 hr	
Clearing	Histoclear	Overnight	
Clearing	Histoclear	2 hrs	
Clearing	Histoclear	2 hrs	
Infiltration	Paraffin Wax	2 hrs	
Infiltration	Paraffin Wax	2 hrs	
Infiltration	Paraffin Wax	Overnight	
Embedding			
Sectioning			

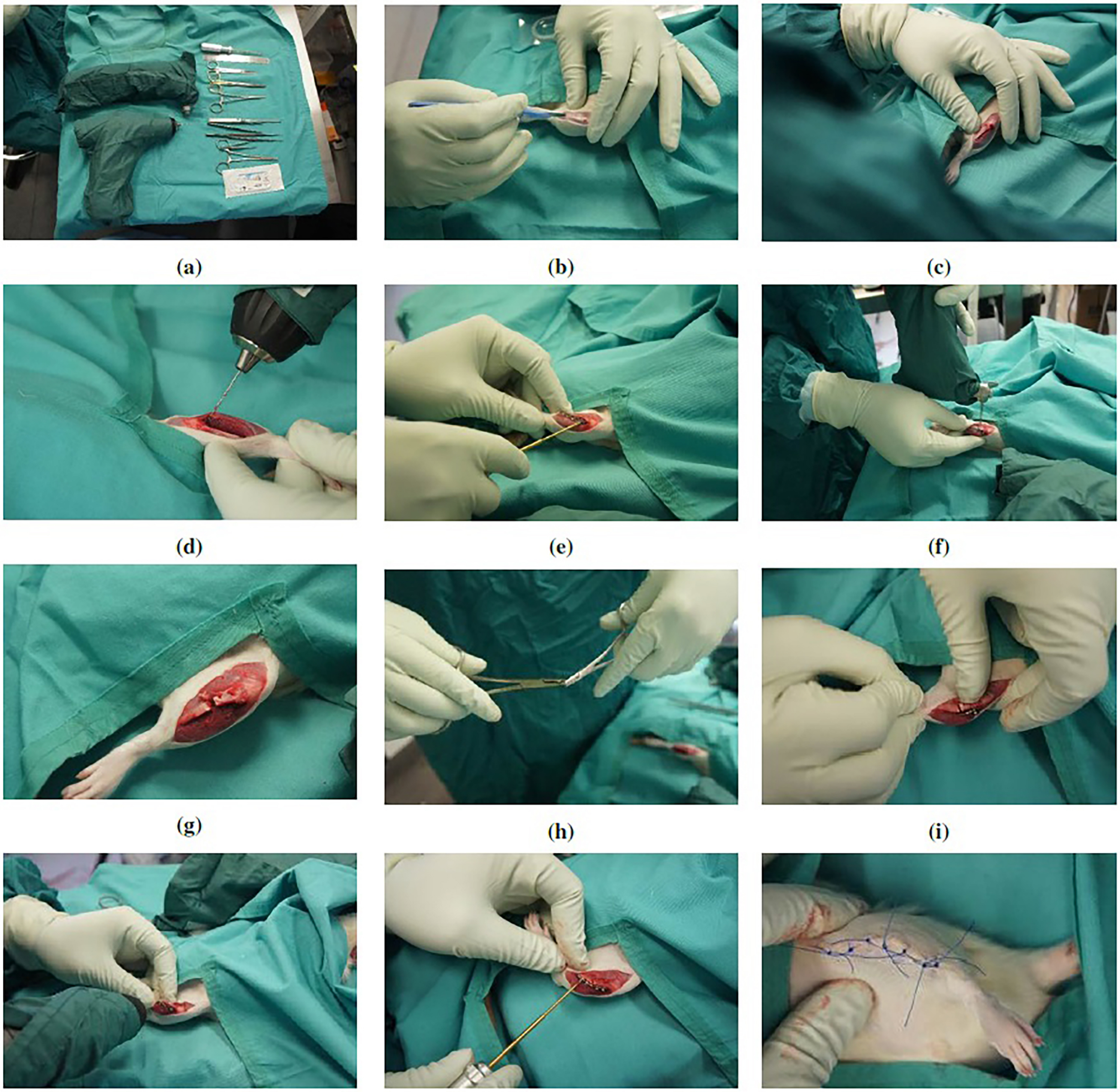

(a) (b) (c) (d) (e) (f) (g) (h) (i) (j) (k) (l)

Figure S1. Surgical technique for inducing a tibial varus osteotomy. (a): Setup of equipment on sterile drape in preparation for surgery. (b): Incision made using scalpel on the frontal side of the shank to expose the tibia. (c): Exposed tibia. (d): Hole drilled for proximal screw placement adjacent to the insertion point of the patella tendon. (e): Screw inserted into the proximal hole in order to provide stability of the plate during sawing. (f): Wedge osteotomy created by removing triangular section of bone with the bone saw at the required osteotomy angle. (g) Frontal view of open wedge osteotomy. (h): Plate bent to appropriate angle using a pair of sterile pliers. (i): Plate reinserted onto the lateral side of the tibia and both parts of the tibia are pressed into the correct angle against the plate. Screws are then placed in the 2 proximal-most holes to secure the plate. (j): Bottom 2 holes are drilled in the tibia through the holes in the plate. (k): Final two screws are inserted into the tibia to secure the osteotomy. (l): Biodegradable sutures used to secure the wound internally followed by application of mattress sutures on the exterior of the wound.
